# 

**Published:** 1856-04

**Authors:** 


EDITED BY

BENJAMIN W. RICHARDSON, M.D.,

LICENTIATE OF THE ROYAL COLLEGE OF PHYSICIANS,
PHYSICIAN TO THE ROYAL INFIRMARY FOR DISEASES OF THE CHEST, AND LECTURER ON PUBLIC HYGIENE AT THE GROSVENOR PLACE MEDICAL SCHOOL.

NATIONAL HEALTH

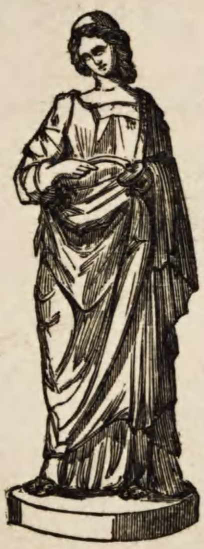

IS NATIONAL WEALTH.

ʽΥΓΙΕΙΑ

*πρεσβίστη μακάρων.*

VOL. II.

PRINTED AND PUBLISHED FOR THE PROPRIETOR BY
THOMAS RICHARDS, 37, GREAT QUEEN STREET.
1856.

WI
J0859m

LONDON
RICHARDS, 37, GREAT QUEEN STREET.

### INDEX.

Aconite, Dr. Pickells on, 378
Adulteration of food, report of committee on, 312
Air and ventilation, 193
Allotments for labouring poor at Southampton, 78
Alvine excretions, fermented, a source of disease, Dr. Routh on, 153
Animal diet, Mr. Moncriff on, *rev.*, 222
Army in the Crimea, report on, 104
Assurance, Dr. Brinton on selection of lives for, *rev.*, 135
Bacon's sanitary philosophy, 126
Bread, composition of, Dr. D. Maclagan on, *rev.*, 31
Burial grounds (Ireland) bill, 101
Canterbury, Mr. Rigden on sanitary state of, 170, 383
Carbon, sulphuret of, poisoning by, 83
Census of Ireland, 311
Charcoal, sanitary applications of, 84
Charitable institutions, diet of, Dr. Routh on, *rev.*, 30
Chloroform, prevention of death from, 29
Cholera, in Oxford, Dr. Acland on, *rev.*, 134; in London, Mr. Simon's report on, 192; and water supply, Dr. Snow on, 239
Coke poisoning in a church, Dr. J. Davy on, 33
Colliers, Mr. W. I. Cox on diseases of, 39, 374
Consumption, pulmonary, Dr. Richardson on hygienic treatment of, 258
Convicts in Australia, diet of, 137
Croydon, mortality of parish of, 76; health of, 411
Dictionary of foods and drinks, 61
Drainage of the metropolis, 411
Dudley, sanitary state of, Mr. Houghton on, 66, 287
England and Wales, pauperism in, 81
Epidemic and endemic diseases, local reports of, 85, 176, 294, 393
First food of infancy, Dr. F. J. Brown on, 59
Food, adulteration of, report of committee on, 312; preservation of, 323
France, hygiene in, 19
Gas stove, a new, 293
Grain and flour imported into Ireland, 81
Hastings, Dr. Greenhill on health and mortality in, 165
Hospitals, Dr. Roberton on ventilation of, *rev.*, 132
Hygiene in France, 19
Hygienic treatment of pulmonary consumption, Dr. Richardson on, 258
Induction in study of disease, 313
Ireland, grain and flour imported into, 81; improved dwellings for labouring classes in, 102; lunatic asylums in, 103; census of, 311
Kaffir doctors, 136
Labouring classes, allotments for at Southampton, 78
——————— in Ireland, bill to encourage improved dwellings for, 102
Lunacy laws, Dr. Webster on, *rev.*, 221
Lunatic asylums in Ireland, 103
Meat, bad, sale of, 311
Medical officers of health, 100; association of in London, report on bad meat, 311
Metropolis local management act, 100
Mining districts, notes on, 26
Mortality in Croydon, 76
Municipal government, its true sphere and duties, 1
Needlewomen, homes for, 82
Nuisances removal bill for Scotland, 100
Nursery government in its sanitary aspects, Dr. H. Barker on, 53, 143, 368
Oxford, Dr. Acland on cholera in, *rev.*, 134
Ozone, 218
Paddington, sanitary condition of, 74
Parasitic developments in the human body, 26, 113

430083

Parliamentary acts, bills, and returns, 100, 191, 312
Patriarchs of Pinner, Dr. Webster on, 223
Pauperism in England and Wales, 81
Physical education of women, 120
Pinner, Dr. Webster on patriarchs of, 223
Poisoning by coke vapour, Dr. J. Davy on, 33; by turpentine vapour, 83; by sulphuret of carbon, *ib.*
Poisons, vegetable, Dr. Pickells on, 378
Post office, second report of Postmaster General on, 102
Preservation of food, 323
Prevention and cure, 8
Preventive medicine, Dr. Barclay on, *rev.*, 221
Prisons, military, report on, 412
Registrar-General's annual report, 412
Registration of births, deaths, and marriages in Scotland, 104
Report on cholera in London, Mr. Simon's, 192; annual, of the Registrar-General, 412; on military prisons, *ib.*
Reports on cities, towns, and districts, 66, 165, 287, 383
——— local, of epidemic and endemic diseases, 85, 176, 294, 393
St. Pancras workhouse, 103
Salt in agriculture, Mr. Northcote on, *rev.*, 32
Sanitary commission of the *Lancet*, 29; legislation, 100, 191, 311, 411; philosophy, of Lord Bacon, 126; reform, in relation to social and moral development, Mr. B. Daniel on, 43; and social science, 66, 165, 287, 383
Scotland, registration of births, deaths, and marriages in, 104
Selection of lives for assurance, Dr. Brinton on, *rev.*, 135
Smoke nuisance, Mr. C. W. Williams on the, *rev.*, 221
State medicine in Great Britain, 105
Stomach, Dr. Turnbull on diseases of, *rev.*, 222
Territorial distribution of population for sanitary purposes, Mr. Rumsey on, 225
Toxicum of the ancients, Dr. Pickells on, 381
Turpentine vapour, poisonous effects of, 83
United States, mortality in cities of, 84
Vaccination, new bill for, 101; Dr. Seaton on protective power of, 343
Vegetable poisons, Dr. Pickells on, 378
Ventilation of hospitals, Mr. Roberton on *rev.*, 132; and air, 193
Water supply in relation to cholera in London, Dr. Snow on, 239
————— of London, 391
Women, physical education of, 120

### TRANSACTIONS

OF THE

### EPIDEMIOLOGICAL SOCIETY

### OF LONDON

### FOR THE YEAR 1856.

LONDON:
T. RICHARDS, 37, GREAT QUEEN STREET.

M.DCCC.LVII.

### BOOKS RECEIVED.

BARKER (T. HERBERT, M.D.) Cystic Entozoa in the Human Kidney. pp. 18. London and Bedford: 1856.

DURNFORD (J. C.), POWER (J. A.), and DANIELL (R. S.) The Correspondence of the *Lancet* Sanitary Commission. pp. 60. London: 1856.

GEORGE (HENRY). An Account of a Mode of Treating the Small-Pox. Fourth Edition. pp. 116. London: 1856.

MACLAGAN (DOUGLAS, M.D., F.R.S.E.) The Composition of Bread. pp. 14. Edinburgh: 1855.

POWELL (R. H., M.D.) Medical Topography of Tunbridge Wells. pp. 157. Tunbridge Wells, and London.

RADCLIFFE (C. B., M.D.) Muscular Contraction a Physical Phenomenon. pp. 23. London: 1856.

THOMSON (SPENCER, M.D.) Outline of Plan for a Compulsory Vaccination Act.

WHITEMAN (R. H.) Address on the Moral and Physical Evils resulting from a Neglect of Sanitary Measures. pp. 28. London: 1856.

WESTALL (E.) Mortality Table of the Parish of Croydon.

WILLIAMS (J. W. H., M.D.) On Unsoundness of Mind in its Medical and Legal Considerations. pp. 238. London, and Dublin: 1856.

[*An excellent treatise, which we shall take an early opportunity of reviewing at length.*]

Second Report of the Post Master General on the Post Office. pp. 84. London: 1856.

The Worth of Fresh Air: No. 1 of Deposits in the Savings' Bank of Wisdom. pp. 31. London: 1856.

Report of the Proceedings of the Sanitary Committee of the Town of Nottingham, for the year ending October 31, 1855. pp. 32. Nottingham: 1855.

---

### IN EXCHANGE.

Scottish Review.
Gazette Médicale de Paris.
Dublin Hospital Gazette.
Literary Gazette.
Glasgow Medical Journal.
Psychological Journal.
Association Medical Journal.
American Journal of the Medical Sciences.
New York Journal of Medicine.
Montreal Medical Chronicle.
Ranking and Radcliffe's Abstract of the Medical Sciences.
Chemist.
Veterinarian.
Charleston Medical Journal.
Edinburgh Medical Journal.
Asylum Journal of Mental Science.
Quarterly Journal of the Chemical Society.

---

*Preparing for publication, price* 1*s.*,

THE VOCATION OF THE MEDICAL SCHOLAR,

BEING THE ANNUAL ORATION DELIVERED BEFORE THE MEDICAL SOCIETY OF LONDON, AT THE EIGHTY-FOURTH ANNIVERSARY, BY

BENJAMIN W. RICHARDSON, M.D.,

Physician to the Royal Infirmary for Diseases of the Chest.

PUBLISHED AT THE REQUEST OF THE SOCIETY.

London: T. Richards, Gt. Queen Street.

### NOTES AND CORRESPONDENCE.

The Editor is anxious to give free scope to the expression of opinion, but does not hold himself responsible for the opinions of those of his contributors who attach their *names* to the articles written by them.

*Reviews.* The Reviews in this Journal are written for the public, not for the special advantage or gratification of authors, however much they may command our respect. The true business of the reviewer is to supply sound information, and it is only a piece of flattery to the writer of a poor work to give it even a severe notice; not to speak of the time that is lost on so useless a task.

*The Nursing Scheme,* adopted and proposed by the Epidemiological Society, in no way comes into collision with other undertakings intended to provide a highly trained class of nurses. On the contrary, it will facilitate all private institutions established for that purpose. The nursing scheme of the society, if adopted at all, must become a national work, and deeply affect the whole of our poorest population, as well as many other classes. As the Earl of Shaftesbury has pledged himself to a numerous deputation, headed by Dr. Babington, to promote the objects of the Committee for Providing the Poor with Nurses, we expect soon to hear that the last impediment, which prevents the realization of the scheme, will be swept away. We again say that the plan we speak of, militates neither against the Nightingale nor any other design. The sooner the former is carried out, the more readily will Miss Nightingale and her friends find material to work with. It will afford us pleasure to have it in our power to announce in our next number, that the Committee which has now been at work for two years, has, with the aid of the Earl of Shaftesbury, succeeded in obtaining the desired order from the Poor Law Board.

*Medical Benevolent Fund.* We are glad to learn that the biennial dinner of this Institution has been fixed for Wednesday the 4th of June at the London Tavern, under the presidency of the Right Honourable Henry Labouchere. We rejoice to give a wider currency to this event; and invite our readers to the support of this fund, as one of the best medical charities.

*Local Registration of Disease.* It will be accepted as a kindness from any of our readers who have not as yet furnished us with local returns of disease, if they will undertake this labour. The sheet for recording observations is simply constructed, gives but little trouble, and will be supplied regularly on application to the Editor.

Dr. Spencer Thomson's suggestion will be remembered.

Papers from Dr. Greenhill, Dr. Powell, W. C. Lake, Esq., and other contributors are in hand, but are of necessity deferred until next number.

